# Immune response profiles of calves following vaccination with live BCG and inactivated *Mycobacterium bovis* vaccine candidates

**DOI:** 10.1371/journal.pone.0188448

**Published:** 2017-11-20

**Authors:** E. M. D. L. van der Heijden, J. Chileshe, J. C. M. Vernooij, C. Gortazar, R. A. Juste, I. Sevilla, J. E. Crafford, V. P. M. G. Rutten, A. L. Michel

**Affiliations:** 1 Department of Infectious Diseases & Immunology, Division of Immunology, Faculty of Veterinary Medicine, Utrecht University, Utrecht, The Netherlands; 2 Bovine Tuberculosis and Brucellosis Research Programme, Department of Veterinary Tropical Diseases, Faculty of Veterinary Science, University of Pretoria, Pretoria, South Africa; 3 Department of Farm Animal Health, Faculty of Veterinary Medicine, Utrecht University, Utrecht, The Netherlands; 4 SaBio Instituto de Investigación en Recursos Cinegéticos IREC (CSIC-UCLM-JCCM), Ciudad Real, Spain; 5 NEIKER-Instituto Vasco de Investigación y Desarrollo Agrario, Animal Health Department, Bizkaia Science and Technology Park, Derio (Bizkaia), Spain; 6 SERIDA, Villaviciosa, Asturias, Spain; 7 National Zoological Gardens of South Africa, Pretoria, South Africa; Public Health England, UNITED KINGDOM

## Abstract

Conventional control and eradication strategies for bovine tuberculosis (BTB) face tremendous difficulties in developing countries; countries with wildlife reservoirs, a complex wildlife-livestock-human interface or a lack of veterinary and veterinary public health surveillance. Vaccination of cattle and other species might in some cases provide the only suitable control strategy for BTB, while in others it may supplement existing test-and-slaughter schemes. However, the use of live BCG has several limitations and the global rise of HIV/AIDS infections has furthermore warranted the exploration of inactivated vaccine preparations. The aim of this study was to compare the immune response profiles in response to parenteral vaccination with live BCG and two inactivated vaccine candidates in cattle.

Twenty-four mixed breed calves (*Bos taurus*) aged 4–6 months, were allocated to one of four groups and vaccinated sub-cutaneously with live *M*. *bovis* BCG (Danish 1331), formalin-inactivated *M*. *bovis* BCG, heat-killed *M*. *bovis* or PBS/Montanide™ (control). Interferon-γ responsiveness and antibody production were measured prior to vaccination and at weekly intervals thereafter for twelve weeks. At nine weeks post-priming, animals were skin tested using tuberculins and MTBC specific protein cocktails and subsequently challenged through intranodular injection of live *M*. *bovis* BCG.

The animals in the heat-killed *M*. *bovis* group demonstrated strong and sustained cell-mediated and humoral immune responses, significantly higher than the control group in response to vaccination, which may indicate a protective immune profile. Animals in this group showed reactivity to the skin test reagents, confirming good vaccine take. Lastly, although not statistically significant, recovery of BCG after challenge was lowest in the heat-killed *M*. *bovis* group.

In conclusion, the parenteral heat-killed *M*. *bovis* vaccine proved to be clearly immunogenic in cattle in the present study, urging further evaluation of the vaccine in challenge studies using virulent *M*. *bovis* and assessment of vaccine efficacy in field conditions.

## Introduction

Control of bovine tuberculosis (BTB), caused by *Mycobacterium bovis* (*M*. *bovis*), is urgently needed on a global scale. The detrimental effects on the cattle industry worldwide as well as on wildlife conservation [1] are noteworthy, with global losses of approximately $3 billion annually [2], and BTB is furthermore of great public health concern. Although “test-and-slaughter” is the conventional control strategy that allowed eradication of the infection in many developed countries, it has proven less effective or unaffordable in other countries facing a multitude of constraints [3, 4], and vaccination of cattle and other species is being considered as a possible alternative approach to BTB control. To date, the only available vaccine is that produced with the Bacille Calmette-Guérin (BCG) strain, which originated from virulent *M*. *bovis* through attenuation and was first used in humans in 1921 [5, 6]. Live BCG is currently registered for use in humans and badgers only [7]. Its use in cattle is prohibited in the EU [8] due to induction of immune responsiveness that interferes with the standard diagnostic methods for BTB utilizing tuberculins and in addition because of the widely variable degree of protection it provides. Hence research efforts have focused on the development of diagnostic reagents that can differentiate between infected and vaccinated animals (DIVA) and on potentiation of the protective effect of BCG [8]. Different strategies using live BCG as either the priming or boosting vaccine in combination with a viral vector [9], recombinant DNA or sub-unit vaccine formulations [10] incorporating various *Mycobacterium tuberculosis* complex (MTBC) specific antigens, have been explored, with conflicting results. Skinner et al. [11] found that the use of a DNA prime-BCG boost regimen using plasmids encoding Hsp65, Hsp70 and Apa was able to significantly improve protection against BTB compared to BCG alone. Likewise, a study by Vordermeier et al. [9], showed that the efficacy of BCG seemed to improve after boosting with a formulation of recombinant attenuated adenovirus expressing antigen 85A. However, vaccination with MPB70 or MPB83 DNA plasmids was not found to be protective in cattle [12].

The rise of the HIV/AIDS pandemic prompted a renewed interest in killed vaccine candidates for protection against tuberculosis [13], as immunocompromised individuals are at risk of developing disseminated disease (BCG-osis) after vaccination with live BCG [10]. This is of special importance in southern Africa, where HIV prevalence is amongst the highest in the world [14]. In these regions, a complex wildlife-livestock-human interface furthermore increases the risk of zoonotic transmission of infectious agents [15]. The use of an inactivated rather than a live vaccine against BTB in cattle, would eliminate the risk of potential propagation of the vaccine strain in food producing animals, a concern raised by the Department of Agriculture, Forestry and Fisheries (DAFF) in South Africa (DAFF, personal communication, November 2014). Vaccination with formalin-inactivated BCG in a Novasome™ adjuvant conferred protection against tuberculosis in guinea pigs [13] and subsequent evaluation in cattle by Whelan et al. [16] demonstrated strong IFN-γ and IgG responses. Promising results of parenteral and oral vaccination with a heat-killed *Mycobacterium bovis* vaccine have since been obtained in several species [17–19]. The aim of the present study was to compare the immune response profiles in response to the parenteral vaccination with live BCG and two inactivated vaccine candidates in cattle. Cell-mediated and humoral immune response profiles resulting from vaccination, skin testing and BCG challenge were monitored over time and compared to a control group.

## Materials & methods

### Animals

This study was carried out in strict accordance with the guidelines of the Animal Use and Care Committee of the University of Pretoria and the protocol was approved (Certificate number V066-15) prior to commencement of the study.

Twenty-four mixed breed *Bos taurus* calves (4–6 months of age; 12 males and 12 females) from a local beef herd with a known BTB free history were used in this study. Exclusion criteria included prior infection with *M*. *bovis* as determined by the bovine IFN-γ release assay (BOVIGAM^®^) and serological testing (IDEXX TB ELISA). All animals were subjected to a full clinical examination, received prophylactic treatment to prevent parasitic and bacterial infections and were allowed to acclimatize to their new environment at the class II biological containment holding facilities of the University of Pretoria Biomedical Research Center (UPBRC) for a period of 6 weeks. The facilities comprised of individual stalls (physical contact between animals in adjacent stalls was not possible) that held the animals of the same vaccine group in pairs, in a spacious, closed and well ventilated cattle holding facility. In view of maturation of the animals in course of the experiment the animals were grouped in pairs based on their sex and each pair was randomly allocated to a pen. Each pen was assigned to one of the treatment groups beforehand. Animals of the four different groups were kept separated.

### Experimental timeline

**Fig 1:**
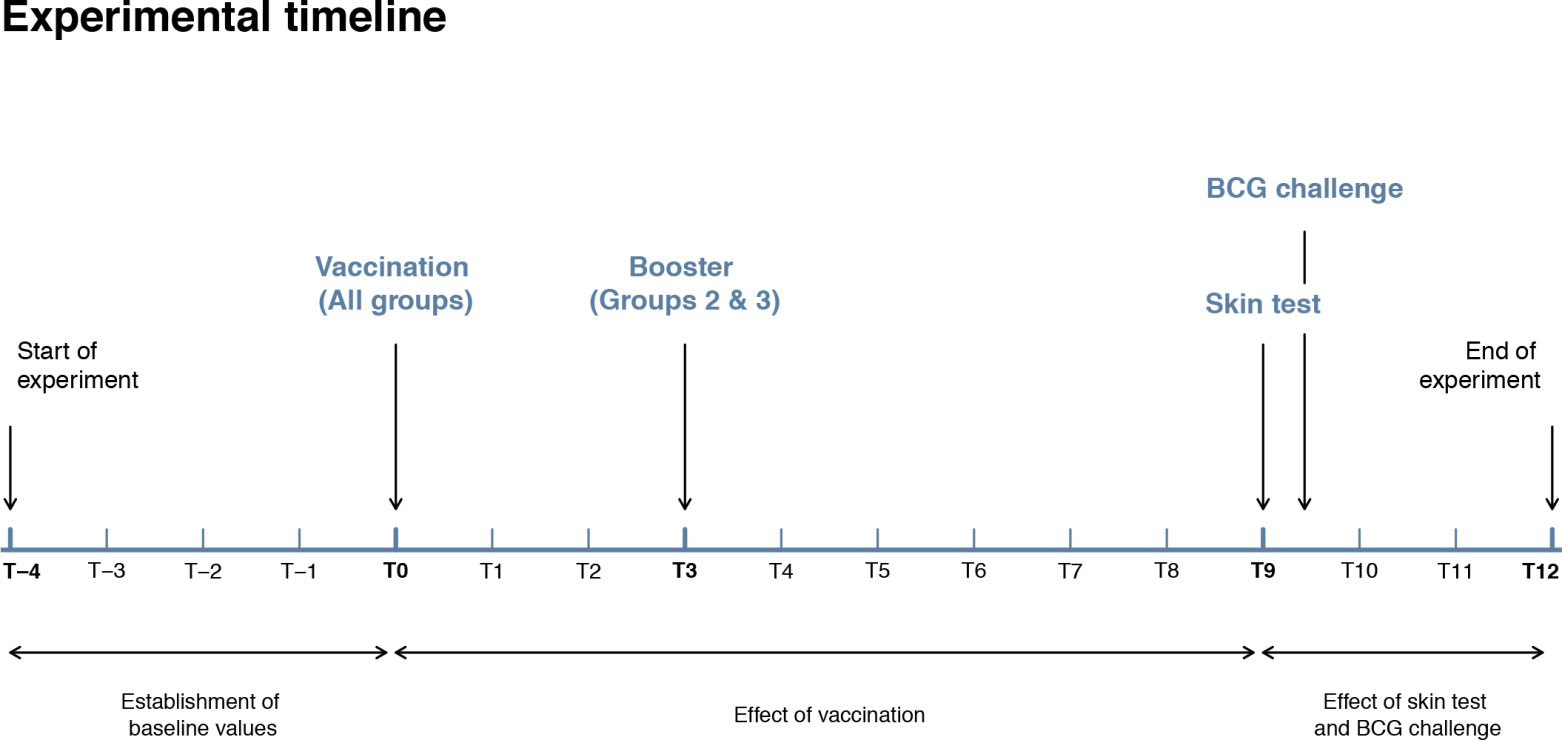
Experimental timeline for the study. Baseline values were established from T-4 until T0 with a sampling interval of 2–3 weeks. After vaccination (T0) until the end of the experiment (T12), the sampling interval was 1 week. Animals were skin tested at T9, challenged with BCG at T9 + 3 days and euthanized at T12. T(x) = time point (week number).

The experimental timeline for the study is depicted in Fig 1.

### Vaccination

Calves were assigned to one of four groups (n = 6 per group). Groups 1–3 received different vaccines and group 4 served as an unvaccinated control group. In groups 2–4, the adjuvant Montanide™ ISA 50 V 2 (SEPPIC, France) was used; a water-in-oil adjuvant, compatible with inactivated antigens, recommended for use in cattle. While Montanide™ stimulates both humoral and cell-mediated immunity, it induces a milder local reaction as compared to other adjuvants (unpublished data).

#### Live *M*. *bovis* Bacille Calmette-Guérin (Group 1)

Lyophilized live *M*. *bovis* BCG Danish 1331 (Statens Serum Institute, Denmark) was reconstituted with the diluent provided by the manufacturer and made up to a concentration of approximately 2 x 10^6^ CFU per ml. One milliliter was administered subcutaneously in the left mid cervical area to all animals in group 1 at T0.

#### Formalin-inactivated *M*. *bovis* Bacille Calmette-Guérin (Group 2)

The formalin-inactivated *M*. *bovis* BCG vaccine was prepared as previously described [13], with a 50% reduction in the formalin treatment time. Briefly, reconstituted live *M*. *bovis* BCG Danish 1331 were suspended in 1.5% formalin (v/v) in phosphate buffered saline (PBS) (Sigma-Aldrich, South Africa). The suspension was stirred continuously at 4°C for 48h and subsequently centrifuged at 14,000 x *g* for 15 minutes at 4°C. The cells were then washed twice, resuspended in sterile PBS to a concentration of 4 x 10^7^ cells/ml and stored at 4°C until use (no longer than three days). Successful inactivation of the BCG was demonstrated by inoculation of aliquots of the vaccine on Löwenstein-Jensen (LJ) slants enriched with pyruvate, followed by incubation for 10 weeks. No growth was observed. The vaccine consisted of formalin-inactivated BCG and Montanide™ ISA 50 V 2 (SEPPIC, France) adjuvant carefully emulsified and made up to a concentration of 2 x 10^7^ cells/ml. One milliliter was administered subcutaneously in the left mid cervical area to all animals in group 2 at T0 and again at T3 (booster).

#### Heat-killed *M*. *bovis* (Group 3)

The heat-killed *M*. *bovis* vaccine was prepared as previously described [17]. The vaccine consisted of heat-inactivated *M*. *bovis* (Neiker strain) and Montanide™ ISA 50 V 2 (SEPPIC, France) adjuvant carefully emulsified and made up to a concentration of 1 x 10^7^ CFU/ml. One milliliter was administered subcutaneously in the left mid cervical area to all animals in group 3 at T0 and again at T3 (booster).

#### Control group inoculum (Group 4)

Phosphate buffered saline (Sigma-Aldrich, South Africa) and 50% Montanide™ ISA 50 V 2 (SEPPIC, France) adjuvant (v/v) were carefully emulsified to serve as an inoculum for the control group. One milliliter was administered subcutaneously in the left mid cervical area to all animals in group 4 at T0.

### Sample collection

Whole blood was collected from the jugular vein using a vacutainer system at several time points prior to vaccination for the BOVIGAM^®^ assay and IDEXX TB ELISA and outcomes were averaged in order to establish baseline values. Thereafter whole blood samples were collected every week for a total of twelve weeks (Fig 1).

### Bovine IFN-γ release assay

Heparinized whole blood samples from all calves were individually processed within 2–3 hrs after collection. Antigen stimulations for the BOVIGAM^®^ assay were carried out in 48-well cell culture plates (Cellstar^®^ Greiner Bio One, Germany) as previously described [20]. At each time point, undiluted heparinized blood was aliquoted into 1 ml per well and stimulated with pokeweed mitogen (PWM; 5 μg/ml) as a positive sample control, PPD-B (purified protein derivative of *M*. *bovis*; 600 IU/ml), PPD-A (purified protein derivative of *M*. *avium*; 1000 IU/ml) and PPD-F (purified protein derivative of *M*. *fortuitum*; 28.5 μg/ml, ARC-Onderstepoort Veterinary Institute) [19]. One aliquot of whole blood was left unstimulated to serve as a negative control. At three time points (T0, T3 and T9), additional aliquots of whole blood were stimulated with the recombinant mycobacterial proteins ESAT-6 (5 μg/ml) and CFP-10 (5 μg/ml) (LIONEX GmbH, Germany). The samples were incubated at 37°C for 20 hrs, after which supernatants were harvested. Interferon-γ detection was carried out according to the manufacturer’s protocol (Thermo Fisher Scientific, South Africa). Criteria for sample validity were an optical density value (OD) ≥ 0.45 for PWM and OD ≤ 0.35 for the negative control (OD_neg_). Responses elicited by TB antigens were corrected by subtracting the OD-value of the negative control (OD_bov_ minus OD_neg_, OD_av_ minus OD_neg_, OD_fort_ minus OD_neg_, OD_ESAT6_ minus OD_neg_ and OD_CFP10_ minus OD_neg_).

### Serology

Blood samples were collected without anticoagulants and left to clot overnight at ambient temperature. Sera were harvested the next day and subsequently tested for the presence of *M*. *bovis* specific antibodies with the IDEXX TB ELISA, using a 1:50 dilution of the samples and controls, according to the manufacturer’s protocol (IDEXX, USA). Criteria for the test validity were an OD ≥ 0.3 for the positive control and OD ≤ 0.2 for the negative control. Sample/positive control (S/P) ratios were calculated according to the manufacturer’s protocol.

### Skin test

At T9, the skin test was performed according to OIE [21] recommendations, with the addition of two protein cocktails alongside the standard tuberculins. Briefly, hair was clipped at 4 sites on the left mid-cervical region that were injected intradermally with 0.1 ml of PPD-B (30,000 IU/ml), PPD-A (25,000 IU/ml), protein cocktail 1 (PC1; containing 10 μg/ml of ESAT-6, CFP-10 and Rv3615c each) and protein cocktail 2 (PC2; containing 10 μg/ml of ESAT-6, CFP-10, Rv3615c and Rv3020c each) [22], respectively. After 72 hrs the injection sites were inspected and palpated for signs of a delayed-type hypersensitivity reaction and the skin fold thickness measured to calculate the difference (Δmm) between pre-injection (0hrs) and post-injection (72hrs) measurements. Differences in increase of skin fold thickness of ≥4 mm between the bovine and avian injection sites and/or the presence of typical clinical signs (necrosis, edema, heat, pain) in combination with a lower increase were considered positive skin test reactions; a difference of 2–4 mm between bovine and avian injection sites was considered a suspect result; while a difference of ≤0 to 2 mm between bovine and avian injection sites was considered a negative skin test. The reactions to the protein cocktails were interpreted according to Jones et al. [22]; an increase in skin fold thickness of ≥ 1 mm was considered positive.

### Intranodular BCG challenge

As an alternative challenge approach, the animals were inoculated in the right prescapular lymph node with live *M*. *bovis* BCG Danish 1331 at T9 + 3 days (after reading of the skin test). This approach has previously been shown to be a viable alternative to challenge with pathogenic *M*. *bovis* without the need for biosafety level 3 facilities [23] to serve as a preliminary evaluation of protection. Briefly, the lyophilized live *M*. *bovis* BCG Danish 1331 vaccine strain was reconstituted in the diluent provided by the manufacturer (Statens Serum Institute, Denmark), inoculated on LJ slants containing pyruvate and incubated for 4 weeks at 37°C. A challenge inoculum was prepared through suspension of the fresh mycobacterial culture in PBS to a concentration of 2 x 10^8^ CFU/ml and 1 ml was injected. At T12 the animals were euthanized by means of a captive bolt and the left (control) and right (inoculated) prescapular lymph nodes were harvested.

### Mycobacterial culture

The weights of the prescapular lymph nodes, collected at T12, were measured to assess inflammation and cellular congestion. Each sample was weighed and inspected for the presence of lesions. In the absence of lesions, a representative sample of ± 2 g from the center of each lymph node (the site of BCG inoculation) was collected aseptically for culture. The samples were homogenized in a final volume of 7 ml sterile distilled water and decontaminated with an equal volume of 2% hydrogen chloride (HCl) during 10 minutes. The samples were then centrifuged at 2550 x *g* for 10 minutes and the supernatant discarded. Subsequently the samples were resuspended in 7 ml of sterile distilled water and centrifuged at 2550 x *g* for 10 minutes, in order to remove remaining HCl. The supernatant was discarded and the pellet resuspended in 3 ml of distilled water and inoculated onto LJ slants containing pyruvate and incubated at 37°C for 10 weeks. Bacterial counts were determined as CFU per gram of lymph node.

Confirmation of mycobacterial growth as being *M*. *bovis* BCG was done using the polymerase chain reaction (PCR) targeting the regions of difference (RD) RD1, RD4 and RD9 as previously described [24].

### Data analysis

Statistical analyses of the data gathered in this study were conducted in R version 3.3.0 [25], as described below.

#### Bovine IFN-γ release assay

A linear mixed effects model [26] was used to analyze the results of the BOVIGAM^®^ assay in the different groups as compared to the control group. For analysis of PPD-B, PPD-B/PPD-A, PPD-B/PPD-F, ESAT-6 and CFP-10, data were log transformed, after adding 0.5, 0.5, 0.75, 0.05 and 0.05 to each value of the outcome variable (to achieve positive values), respectively, to meet the model assumptions of normality and homoscedasticity. Explanatory variables were time, vaccination group and the interaction between both, which proved to be the final model. A variance function was added to the models of PPD-B, ESAT-6 and CFP-10 to allow for different standard deviations in the vaccination groups. Correlated observations within animals were accounted for by using a random intercept and slope for the animal ID. The Akaike Information Criterion (AIC) was used to select the best model.

#### Serology

A linear mixed effects model [26] was used to analyze the results of the IDEXX TB ELISA in the different groups as compared to the control group. For analysis of the S/P ratio data were log transformed, after adding 0.1 to each value of the outcome variable (to achieve positive values), to meet the model assumptions of normality and homoscedasticity. The other criteria and parameters of the model were the same as for the model of PPD-B.

#### Skin test

In order to account for the heteroscedasticity of the variances, a double generalized linear model [27] was used to analyze the skin reactions to the tuberculins (ΔPPD-B minus ΔPPD-A in mm). A simple general linear model was used to analyze the skin reactions to the protein cocktails (ΔPC1 and ΔPC2 in mm). The explanatory variable was vaccination group.

#### Intranodular BCG challenge

Prescapular lymph node weights in the treatment groups were compared to the control group using a linear mixed effects model [26]. The outcome variable was log transformed to meet the model assumptions of normality and homoscedasticity. Explanatory variables were lymph node side, vaccination group, the interaction between both and gender which proved to be the final model. Correlated observations within animals were accounted for by using a random intercept and slope for the animal ID.

A negative binomial generalized linear model was used to compare the bacterial counts from the right prescapular lymph nodes of the treatment groups to the control group. The explanatory variable was vaccination group.

## Results

### Bovine IFN-γ release assay

**Fig 2:**
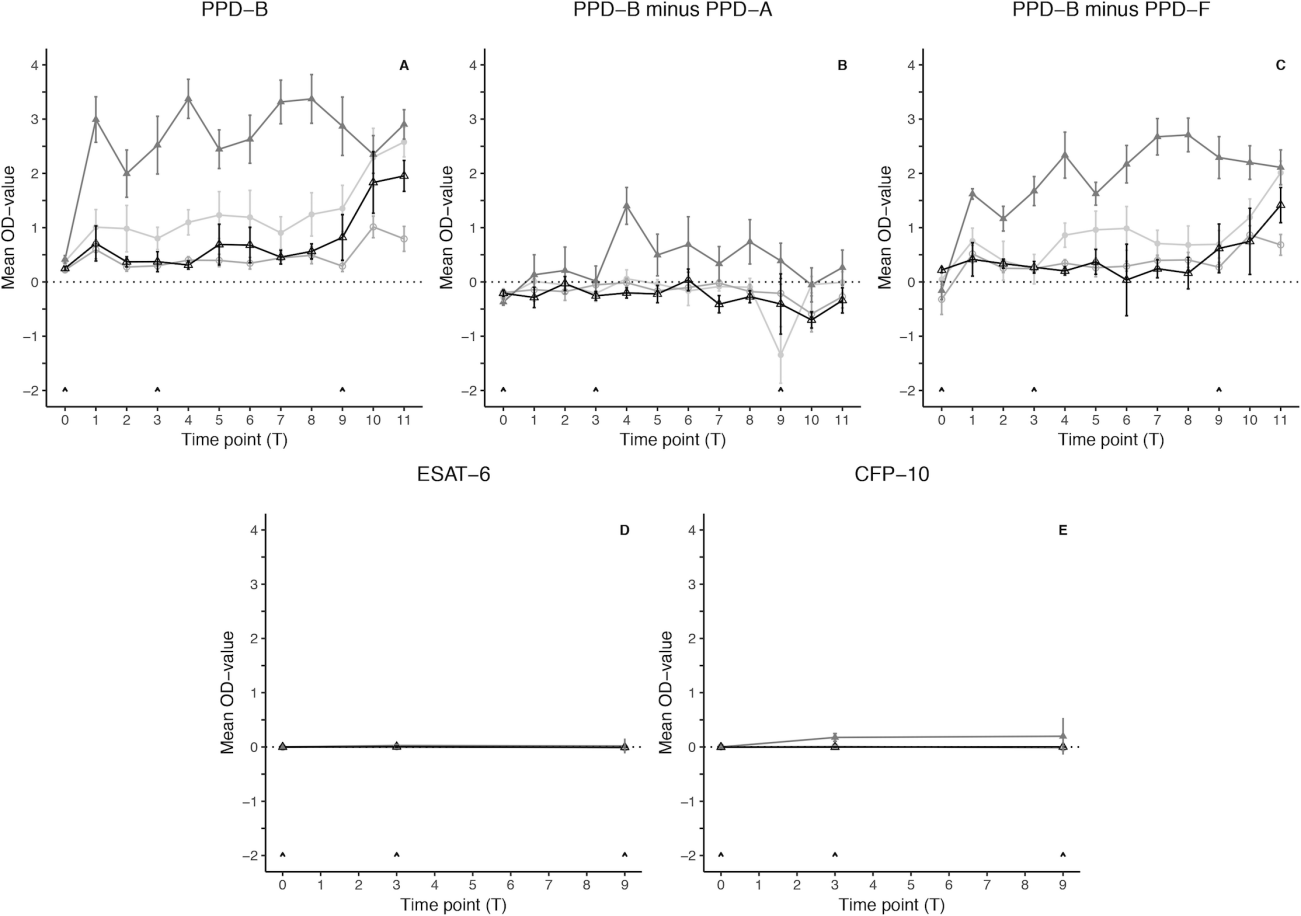
Interferon-*γ* responses in the BOVIGAM^®^ assay. Mean OD-values in response to (A) PPD-B, (B) PPD-B minus PPD-A, (C) PPD-B minus PPD-F, (D) ESAT-6 and (E) CFP-10. ● = Live *M*. *bovis* BCG; ○ = Formalin-inactivated BCG; ▲ = Heat-killed *M*. *bovis*; △ = Control group. Error bars indicate the standard error of the mean (±SEM) for each time point. Arrows at T0, T3 and T9 indicate priming vaccination, booster vaccination and SICTT and BCG challenge, respectively.

Cell-mediated immunity (CMI) as a consequence of vaccination with the different vaccines was monitored by means of IFN-γ responses using the BOVIGAM^®^ assay for nine weeks (T1-T9) in all animals and compared to the control group. Interferon-γ responses following skin test and BCG challenge (at T9 and T9 + 3 days, respectively) were monitored for two weeks (T10-T11). One animal from the formalin-inactivated BCG group was excluded from this analysis due to unresponsiveness of white blood cells to stimulation with PWM from T1 to T9. The PPD-B specific IFN-γ responses (corrected for the OD_neg_), expressed as OD values (OD_bov_), in the four treatment groups over time are presented in Fig 2A. The response to PPD-B was analyzed using a linear mixed effects model [26] and compared to the control group (Table A in S2 Dataset). The mean (n = 6) responses to PPD-B prior to vaccination (T0) were below an OD-value of 0.5 for all groups. Mean OD-values for PPD-B in the control group ranged from 0.311 to 0.820 after vaccination (T1-T9). A similar trend was observed in the formalin-inactivated BCG group, with slightly lower mean OD_bov_ values ranging from 0.275 to 0.594 and no significant differences to the control group. The live BCG group showed slightly elevated mean OD_bov_ values as compared to the control group in response to vaccination (T1-T9), ranging from 0.802 to 1.353, and differences were significant at T4 only (Table A in S2 Dataset). The heat-killed *M*. *bovis* group showed the highest OD_bov_ values after vaccination, ranging from 1.996 to 3.374, peaking at T4, and significant differences compared to the control group were observed from early on in the experiment and were sustained up to and including T9 (Table A in S2 Dataset). After skin test and BCG challenge, the mean OD_bov_ values were elevated as compared to T9 in all groups except the heat-killed *M*. *bovis* group. In the control group, mean OD_bov_ values ranged from 1.833 to 1.953. No significant differences to the control group were found in the live BCG and heat-killed *M*. *bovis* groups, with mean OD_bov_ values ranging from 2.296 to 2.578 and 2.349 to 2.903, respectively. In the formalin-inactivated group OD_bov_ values ranged from 0.793 to 1.014 and this was significantly lower as compared to the control group at T11 (Table A in S2 Dataset).

Reactivity to two predominant environmental mycobacteria (*M*. *avium* and *M*. *fortuitum*) was taken into account in the analysis of the IFN-γ responses and presented as OD_bov_-OD_av_ and OD_bov_-OD_fort_ (both corrected for the OD_neg_) in Fig 2B and 2C, respectively. The ratios of PPD-B/PPD-A and PPD-B/PPD-F were analyzed using a linear mixed effects model [26] and compared to the control group (Table A in S2 Dataset). In the PPD-B/PPD-A model, both the live and the formalin-inactivated BCG vaccination groups showed no significant differences when compared to the control group. In the heat-killed *M*. *bovis* group, however, responses significantly higher as compared to the control group were observed at several time points (T4-T9) after vaccination (Table A in S2 Dataset). In the PPD-B/PPD-F model, the immune responses in the live and formalin-inactivated BCG groups were largely comparable to those in the control group, but differences significantly higher as compared to the control group were seen in the live BCG group at T6 (Table A in S2 Dataset). Again, the heat-killed *M*. *bovis* group showed significantly higher responsiveness as compared to the control group at multiple time points (T1 and T3-T9) after vaccination as well as after skin test and challenge (T10) (Table A in S2 Dataset).

In addition to the classical PPDs as stimulating antigens in CMI testing, ESAT-6 and CFP-10, two antigens widely used in TB research and diagnosis, as they are assumed to be specific for the MTBC, were included in the BOVIGAM^®^ assay at T0, T3 and T9. Fig 2D and 2E present the IFN-γ responses specific to these antigens in the four vaccination groups during the course of the experiment. The responses to ESAT-6 and CFP-10 were analyzed using a linear mixed effects model [26] and compared to the control group (Table B in S2 Dataset). One animal in the heat-killed *M*. *bovis* group showed an extremely high value at week 9 and this entry was excluded from the model. Mean OD-values for ESAT-6 (OD_ESAT6_) in the control group ranged from -0.026 to 0.064. Similar trends were observed in the other vaccination groups with OD _ESAT6_ values ranging from -0.034 to 0.011 (live BCG), from -0.049 to 0.022 (formalin-inactivated BCG) and from -0.019 to 0.094 (heat-killed *M*. *bovis*). There were no significant differences in the responses to ESAT-6 in any of the vaccination groups as compared to the control group (Table B in S2 Dataset). Mean OD-values for CFP-10 (OD_CFP10_) in the control group ranged from -0.021 to 0.016. Similar trends were observed in the vaccination groups receiving the live and formalin-inactivated BCG vaccines with OD_CFP10_ values ranging from -0.023 to 0.036 and from -0.047 to 0.034, respectively. The mean response to CFP-10 in the formalin-inactivated BCG group was significantly lower as compared to the control group at T9 (Table B in S2 Dataset). Mean OD_CFP10_ values in the heat-killed *M*. *bovis* group showed a slight increase as compared to the control group ranging from -0.008 to 0.462 at T3 and from 0.001 to 0.557 at T9, and these were significantly different (Table B in S2 Dataset).

### Serology

**Fig 3:**
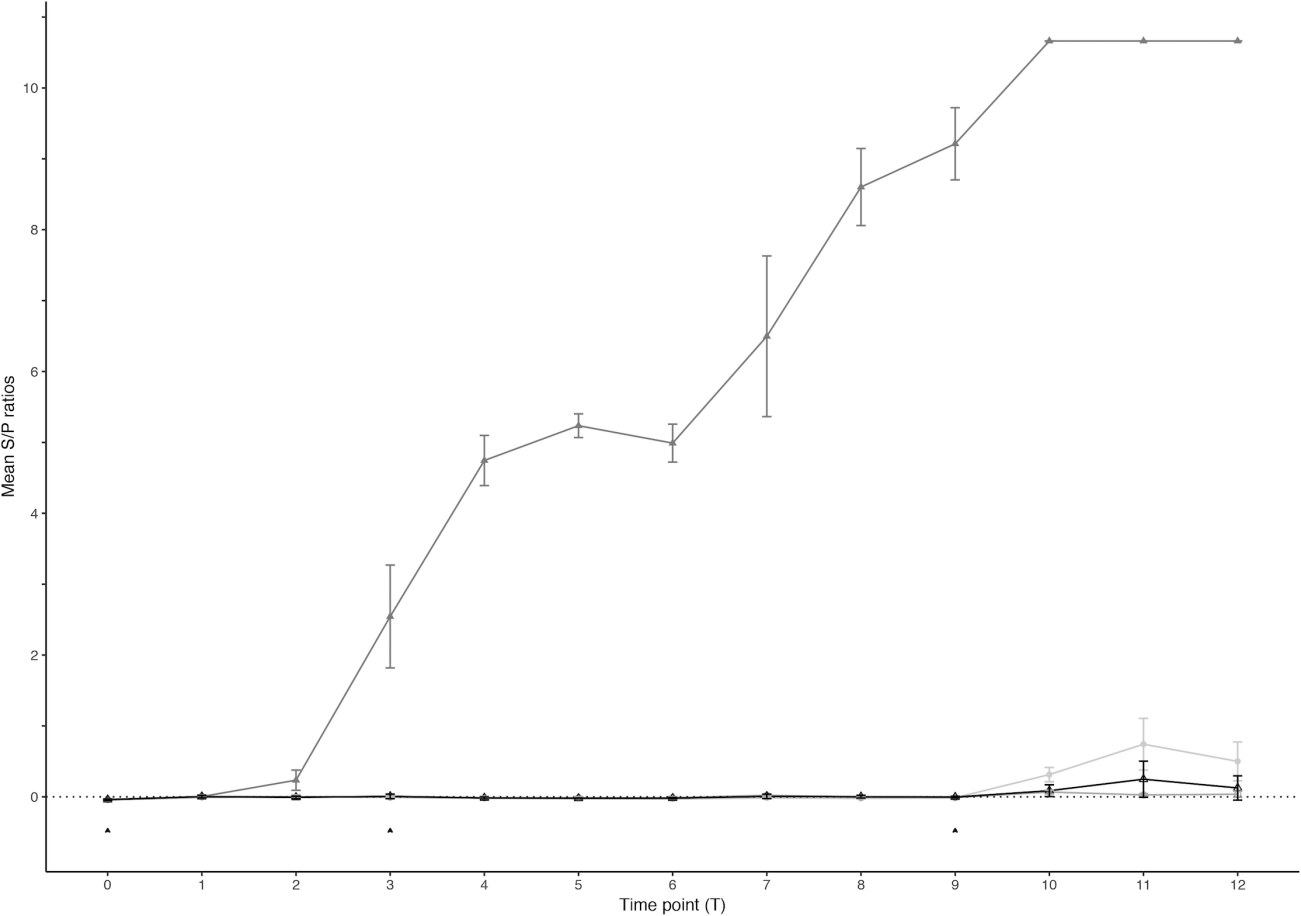
Antibody responses in the IDEXX TB ELISA. Mean sample/positive (S/P) ratios. ● = Live M. bovis BCG; ○ = Formalin-inactivated BCG; ▲ = Heat-killed M. bovis; △ = Control group. Error bars indicate the SEM for each time point. Arrows at T0, T3 and T9 indicate priming vaccination, booster vaccination and SICTT and BCG challenge, respectively.

Humoral immune (HI) responsiveness to the different vaccine candidates was monitored for nine weeks (T1-T9) in all animals and compared to the control group. Responses following skin test and BCG challenge (at T9 and T9 + 3 days, respectively) were monitored for further three weeks (T10-T12). Fig 3 presents the mean S/P ratios for the vaccination groups over time. The S/P ratios were analysed using a linear mixed effects model [26] and compared to the control group (Table C in S2 Dataset). Prior to vaccination, the mean S/P ratios in all groups were approximately -0.04. Mean S/P ratios in the control group ranged from -0.022 to 0.007 after vaccination (T1-T9). Humoral responses after vaccination in the groups receiving the live and formalin-inactivated BCG preparations mirrored those in the control group (Fig 3) and thus no significant differences to the control group were found (Table C in S2 Dataset). In the heat-killed *M*. *bovis* group, a humoral response was detected from as early as T2, which increased and was sustained during the course of the experiment (Fig 3), with values ranging from 0.003 to 9.213 after vaccination (T1-T9). The S/P ratios in this group were found to be significantly higher compared to those in the control group at several time points (T2-T9) (Table C in S2 Dataset). After skin testing and BCG challenge (at T9 and T9 + 3 days, respectively), mean S/P ratios in the control group showed a very slight increase, ranging from 0.086 to 0.245. In contrast, there appeared to be no response to skin test and BCG challenge in the formalin-inactivated BCG group and S/P ratios ranged from 0.027 to 0.067. The S/P ratios observed in the live BCG group at time points T10 to T12 were significantly higher than in the control group (Table C in S2 Dataset). The S/P ratios in the heat-killed *M*. *bovis* group plateaued at a value of 10.663 after skin test and BCG challenge and these values were significantly higher than the control group (Table C in S2 Dataset). The plateau effect was considered a consequence of the ELISA reader’s upper detection limit rather than a true reflection of the optical density.

### Skin test

**Fig 4:**
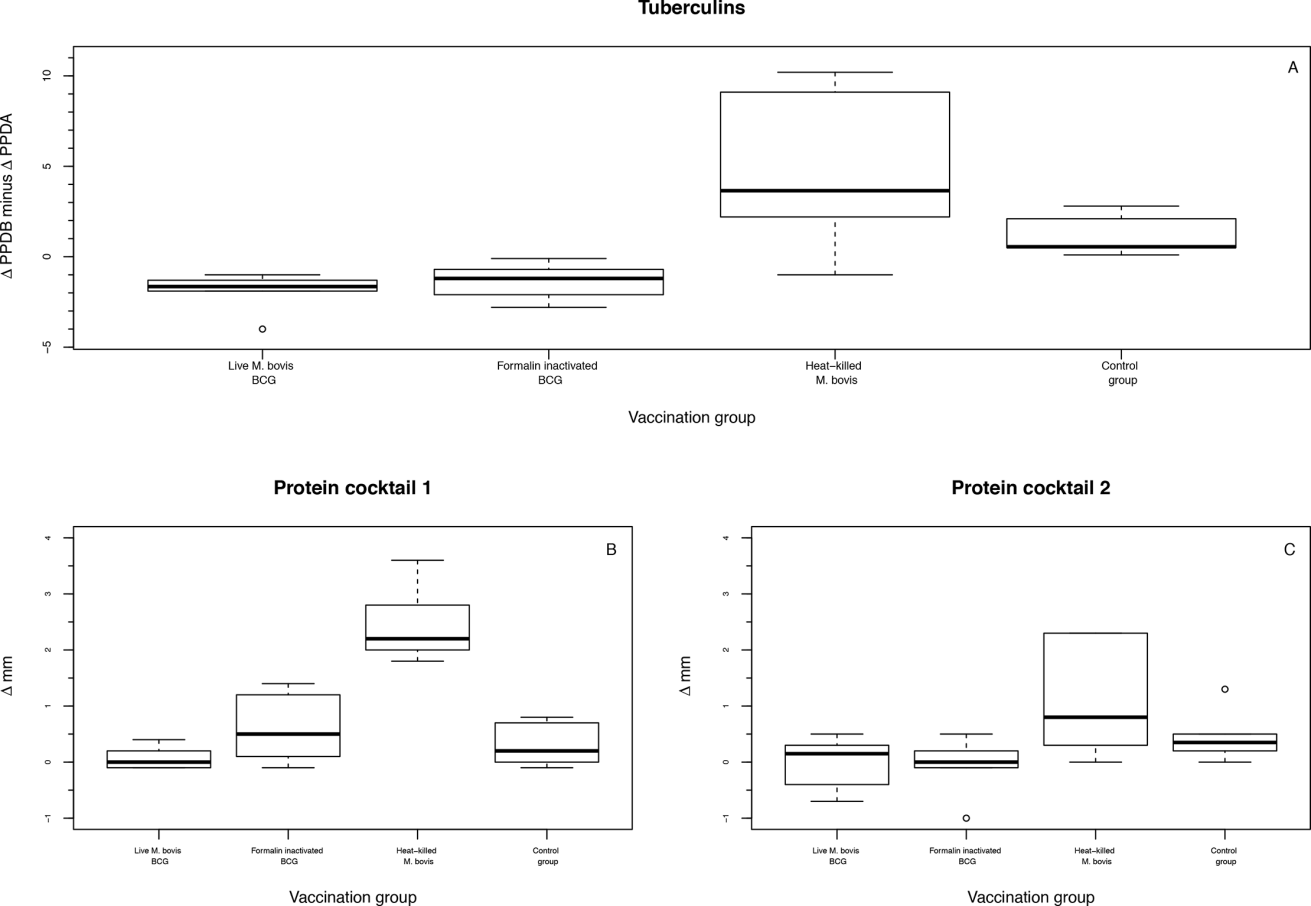
Results of the skin test. Differences in skin fold thickness (A) between PPD-B and PPD-A, (B) of protein cocktail 1 and (C) of protein cocktail 2. ΔPPDB is the difference in skin fold thickness at the PPD-B injection site between 72 hrs (post injection) and 0 hrs (pre-injection). ΔPPDA is the difference in skin fold thickness at the PPD-A injection site between 72 hrs (post injection) and 0 hrs (pre-injection). Δmm is the difference in skin fold thickness of the injection site between 72 hrs (post injection) and 0 hrs (pre-injection).

The skin test was carried out on all animals in all groups. The difference between the increase in skin fold thickness in reaction to PPD-A was deducted from that to PPD-B and presented in Fig 4A, while the reactions to the two protein cocktails PC1 and PC2 are presented in Fig 4B and 4C. A double generalized linear model was used to analyze the reaction to the tuberculins (Table D in S2 Dataset), whereas the reactions to the PC1 and PC2 were analyzed using a simple general linear model (Table E in S2 Dataset).

The difference in increase of skin fold thickness in reaction to the tuberculins (Δmm of PPD-B minus Δmm PPD-A) ranged from -4.0mm to -1.0mm in the control group and as such all animals in this group were characterized as negative reactors. Animals in the formalin-inactivated BCG group showed similar reactions to the PPDs as the control group (differences in increase of skin fold thickness ranged from -2.8mm to -0.1mm), and no significant differences as compared to the control group were found and all animals were characterized as negative reactors (Table D in S2 Dataset). In the live BCG group, the differences in increase of skin fold thickness ranged from 0.1mm to 2.8mm, and these reactions were found to be significantly higher as compared to the control group (Table D in S2 Dataset). In this group, 1/6 animals was classified as a positive reactor (Δmm of 2.8 and edema) and 1/6 animals was classified as a suspect reactor. In the heat-killed *M*. *bovis* group the differences in increase of skin fold thickness ranged from -1.0mm to 10.2mm, and these reactions were found to be significantly higher as compared to the control group (Table D in S2 Dataset). In this group, 3/6 and 2/6 animals were classified as positive and suspect reactors, respectively.

No animals in the control group showed reactions to PC1 and PC2 and the differences (Δmm 72 hrs and 0 hrs) ranged from -0.1mm to -0.4mm and from -0.7mm to 0.5mm, respectively, and animals were characterized as negative reactors. In the live and formalin-inactivated BCG groups, reactions to PC1 and PC2 were slightly elevated as compared to the control group, but no significant differences to the control group were found (Table E in S2 Dataset). In the heat-killed *M*. *bovis* group, the reactions were significantly elevated as compared to the control group and the differences in skin fold thickness ranged from 1.8mm to 3.6mm for PC1 and from 0.0mm to 2.3mm for PC2 (Table E in S2 Dataset). In this group, 6/6 and 3/6 animals were classified as positive reactors to PC1 and PC2, respectively.

### Intranodular BCG challenge

Overall, the weights of the untreated left prescapular lymph nodes (PLNs) showed less variability as compared to those of the right prescapular lymph nodes, which were inoculated with BCG at T9 + 3 days. In the control group the median and interquartile range (IQR) of the left and right prescapular lymph nodes reflect this pattern (median PLN_left_ = 15.29g; IQR PLN_left_ = 3.67g; median PLN_right_ = 14.49g; IQR PLN_right_ = 6.18g). The lymph nodes of the animals in the live BCG group, however, showed lower weights and less variability (median PLN_left_ = 14.14g; IQR PLN_left_ = 1.65g; median PLN_right_ = 13.96g; IQR PLN_right_ = 1.57g). In the formalin-inactivated BCG group the median and interquartile range was slightly elevated compared to the control group (median PLN_left_ = 16.58g; IQR PLN_left_ = 3.20g; median PLN_right_ = 16.14g; IQR PLN_right_ = 5.67g). The weights of the PLNs were highest and showed the most variability in the heat-killed *M*. *bovis* group (median PLN_left_ = 18.42g; IQR PLN_left_ = 3.69g; median PLN_right_ = 16.29g; IQR PLN_right_ = 8.06g). No significant differences to the control group were found for either lymph node side in any of the groups when analyzed using a linear mixed effects model [26] (Table F in S2 Dataset).

### Culture and *M*. *bovis* PCR

Growth of mycobacteria was not observed in cultures of any of the left prescapular lymph nodes and these were omitted from this analysis. Mycobacterial growth was detected in cultures of the right prescapular lymph nodes of 3/6 animals in the live BCG group, 5/6 animals in the formalin-inactivated BCG group, 5/6 animals in heat-killed *M*. *bovis* group and 2/6 of the control animals.

**Fig 5:**
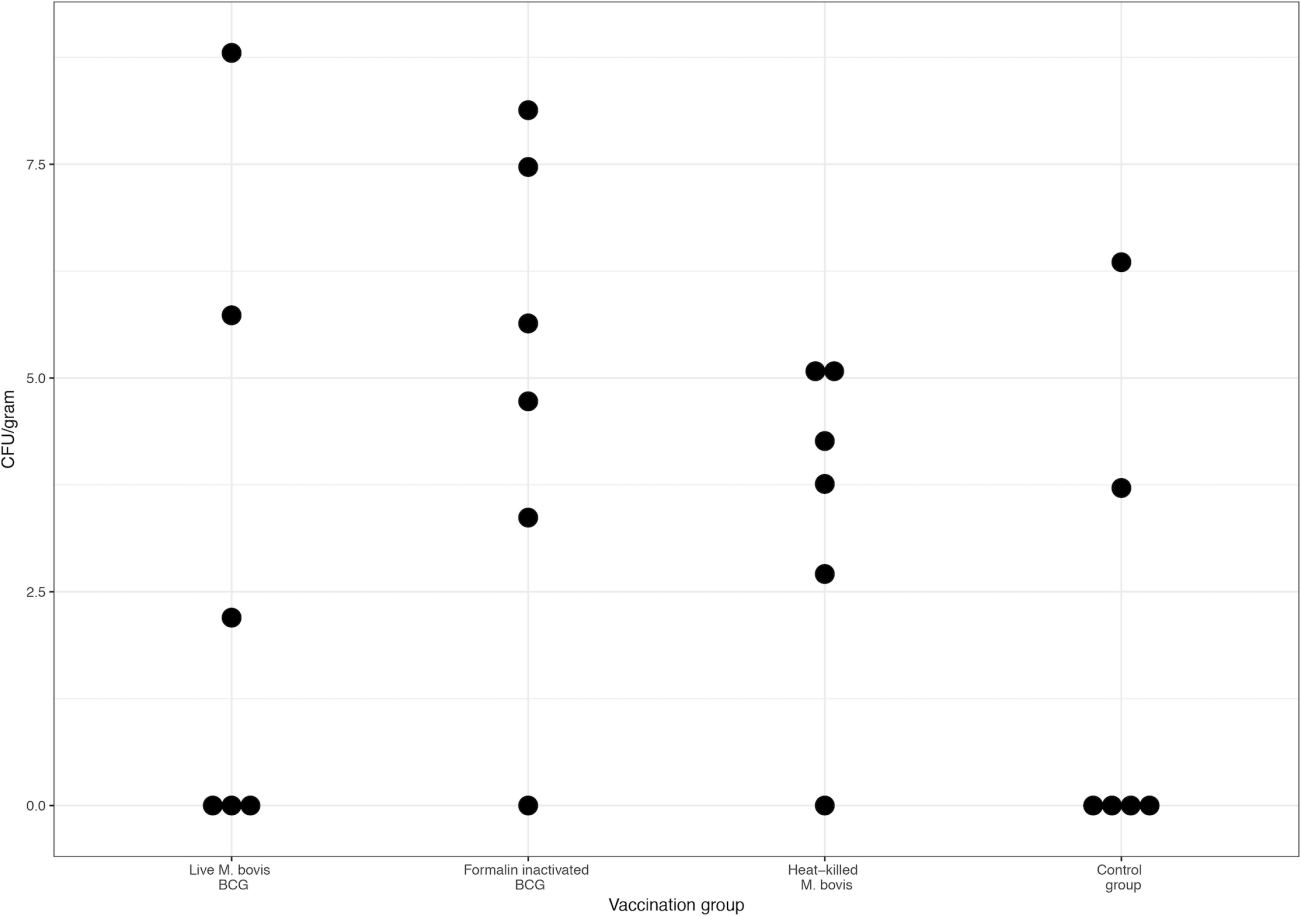
Log transformed bacterial counts (CFU/gram) of the right prescapular lymph nodes. The data was log transformed to accommodate for the large variance. As several lymph nodes had shown no growth, equal to bacterial count = 0, +1 was added to all values to allow for log transformation.

PCR confirmed presence of *M*. *bovis* BCG in all isolates (S1 Fig). In the right prescapular lymph nodes, the highest bacterial counts (CFU/gram) were found in the live BCG group (range = 0–6650), followed by the formalin-inactivated BCG group (range = 0–3402), the control group (range = 0–574) and the heat-killed *M*. *bovis* group (range = 0–182) (Fig 5). The heat-killed *M*. *bovis* group was found to have lower bacterial counts (estimate ratio = 0.730) as compared to the control group, but this difference was not significant (Table G in S2 Dataset). Additionally, although bacterial counts were higher in the live BCG group (estimate ratio = 11.345) and formalin-inactivated BCG (estimate ratio = 9.075) group, these were not significantly different to the control group (Table G in S2 Dataset).

## Discussion

The present study aimed at characterization and evaluation of immune response profiles induced in calves by inactivated *Mycobacterium bovis* vaccine candidates as compared to live BCG and a control inoculum. This immunogenicity study is an important step in the process of evaluation of a new vaccine candidate, before execution of controlled field trials, to assess protective efficacy against BTB in cattle.

The live BCG and formalin-inactivated BCG groups did not exhibit pronounced CMI responses. In the heat-killed *M*. *bovis* group, however, strong and sustained IFN-γ responses to PPD-B were detected that, even when accounting for possible cross-reactivity due to exposure to non-tuberculous mycobacteria (Fig 2A–2C), were significantly higher as compared to the control group. Furthermore, an elevated response to CFP-10, significantly different to the control group, in the heat-killed *M*. *bovis* group at T3 and T9, pointed to a cell-mediated immune response believed to be specific to species of the MTBC. Surprisingly, no response to ESAT-6 was detected in this group. Since CFP-10 and ESAT-6 are found on the region of difference 1 (RD1) gene region of *M*. *bovis* [28] and are co-transcribed [29], it was expected that animals in the heat-killed *M*. *bovis* group would show reactivity, of comparable magnitude, to both proteins. However, heat-inactivation of the *M*. *bovis* strain used in this vaccine at 80°C for 30 minutes [17], may have affected these proteins differently and could explain the lack of a response to ESAT-6. Although the exact underlying processes remain unclear, IFN-γ release in response to *M*. *bovis* specific antigenic stimulation after vaccination is generally accepted to play an important role in the protective mechanism against BTB, emphasizing the role of cell-mediated immunity. This role was confirmed in an ‘ex vivo’ experiment carried out by Juste et al. [19] which determined that bovine macrophages trained with the heat-killed *M*. *bovis* vaccine through intramuscular injection exhibited an increased lytic capacity, which was furthermore proposed as an underlying mechanism of vaccine protection. Moreover, a study in wild boar confirmed that high IFN-γ responses to PPD-B after vaccination with heat-killed *M*. *bovis* are likely to contribute to the protective mechanisms against BTB [17, 30]. Likewise, the CMI responses detected in the heat-killed *M*. *bovis* group in the present study, may point towards a similar protective mechanism in cattle.

In the present study, an early and progressively increasing humoral immune response to the heat-killed *M*. *bovis* vaccine, significantly different from the control group, was found as well (Fig 3). There was no evidence of an HI response in the live BCG and formalin-inactivated groups. The role of humoral immunity in protection against bovine tuberculosis and as a parameter for vaccine efficacy is not particularly well understood and previous studies have demonstrated conflicting results. Wedlock et al. [12] described that significant humoral responses to a MPB70 DNA and protein vaccine regimen could not confer protection in cattle. In contrast, studies using inactivated BCG preparations in mice by Haile et al. [31] illustrated significant antibody responses and their vaccine was more efficacious than live BCG. The importance of an antibody response in mycobacterial infections has furthermore been highlighted in a study evaluating post-exposure vaccination against paratuberculosis and a correlation with vaccine efficacy was established [32]. The heat-killed *M*. *bovis* vaccine elicited antibody responses in wild boar that were suggested to be associated with protection against BTB [17, 30]. The strong HI response detected in the present study could potentially play a role in a protective mechanism against mycobacteria.

The IFN-γ response to stimulation with PPD-B increased in both BCG groups as well as the control group after skin test and BCG challenge. As strong post-challenge IFN-γ responses are commonly associated with a higher lesion burden and lower vaccine efficacy against bovine tuberculosis, previously demonstrated by Hope et al. [33], the present findings may suggest that these vaccinates would not be protected. In contrast, in the heat-killed *M*. *bovis* group an initial decline followed by resurgence of the IFN-γ response was seen from T10-T11, but it remained below levels observed prior to T10. The fact that post-challenge IFN-γ responses were lowest in this group, strengthens the proposition that the heat-killed *M*. *bovis* vaccine appears to elicit a CMI response which may confer protection against BTB in cattle. In tuberculous pleuritis in humans, localized rather than systemic action of the cell-mediated immune response, associated with higher proportions of IFN-γ secreting lymphocytes in the pleural cavity as compared to the blood, has been demonstrated by several research groups [34, 35] and is suggested to be responsible for mycobacterial clearance without therapeutic intervention [36]. The decline in PPD-B specific CMI responses in peripheral blood after challenge in the heat-killed *M*. *bovis* group, could possibly be a result of homing of memory T cells to the site of the challenge, depleting such cells from the periphery. Evaluation of lymphocytes present in the inoculated prescapular lymph nodes would be required to test this hypothesis and was beyond the scope of the current study. Assessment of the weights and bacterial counts of these lymph nodes did, however, show that the weight distribution was the most variable in the heat-killed *M*. *bovis* group, while the bacterial counts were lowest in this group. These findings might potentially be a result of variable influx of T-cells and subsequent increased bactericidal activity.

Animals in the control group showed no reactivity to any MTBC specific reagents used in the skin test, confirming that these animals had not been vaccinated with or exposed to *Mycobacterium bovis*. Skin test reactivity to the tuberculins and protein cocktails in the heat-killed *M*. *bovis* group was significantly different to the control group, clearly indicating the immunogenicity of this candidate. The slightly lower significance found in this group compared to that of the live BCG group can be explained by the greater variance that was found in the differences in skin fold thickness in the heat-killed *M*. *bovis* group. The skin test is a highly important and widely-used diagnostic tool for bovine tuberculosis. Live BCG vaccination is known to interfere with diagnostic tests based on the use of tuberculin [37] and although in our study the immunogenicity of live BCG appeared to be low, reactivity to bovine and avian tuberculin in this group was significantly higher as compared to the control group. The protein cocktails, however, contain known immunogenic proteins both present in (ESAT-6 and CFP-10) or dependent on (Rv3615c) the RD1 region of *M*. *bovis* which is deleted from BCG [38, 39], or proteins present in both *M*. *bovis* and BCG but with differential recognition (Rv3020) [40] and have been developed with the aim of establishing a DIVA principle for BTB [22]. As expected, skin test reactions in the live and formalin-inactivated BCG groups were negligible, whereas the heat-killed *M*. *bovis* vaccine group showed significant skin test reactivity to the tuberculins as well as the protein cocktails. It is interesting to note that although all animals tested positive to PC1, only 3/6 tested positive to PC2. Jones et al. [22] described that the addition of Rv3020 to the cocktail of ESAT-6, CFP-10 and Rv3615c (PC1) increased sensitivity whilst preserving specificity. In the present study, however, it appears that the sensitivity of PC2 is much lower than that of PC1 and it is unclear what the cause of this discrepancy is.

There was no change in the humoral response post-skin test and -challenge in the control group. As the experiment was terminated 3 weeks post-challenge, this is not surprising because the antibody response to mycobacterial infection is known to be a marker of chronic or late-stage of disease [41]. This, in combination with the low immunogenicity of this vaccine as demonstrated in the current study, might also explain the lack of a response in the formalin-inactivated BCG group. There was a moderate antibody response in the live BCG group, which could indicate that priming of these animals to *M*. *bovis* might have been successful, but it is questionable whether this would have been protective. Animals in the heat-killed *M*. *bovis* group appeared to respond to the skin test and BCG challenge with a rapid deployment of *M*. *bovis* specific antibodies as indicated by a marked increase in S/P ratio after T9, which is indicative of clear sensitization to *M*. *bovis* through the initial vaccination and booster vaccine and might be suggestive of a protective response.

### Recovery of BCG after challenge

The greater variability that was found in the right prescapular lymph node weights compared to those of the left PLNs can be explained by the fact that the challenge was performed through intranodular injection into the right PLN, which showed transient visible swelling and tenderness post-injection, whereas there was no intervention in the left PLN. It is interesting to note that the greatest variability of PLN weights was found in the heat-killed *M*. *bovis* group. The meaning of this is not certain and could be due to increased activity of the immune system clearing up infection, or inflammation due to the presence of live mycobacteria. To further investigate the outcome of the challenge, all PLNs were processed for mycobacterial culture. The fact that *M*. *bovis* BCG could not be detected in any of the left PLNs, indicates that mycobacteria were not disseminated throughout the host, in line with the rationale proposed by Villarreal-Ramos et al. [23]. The recovery of BCG from the right PLNs proved more challenging than expected as the right PLNs of 4/6 of the control animals did not yield BCG on culture and 2/6 in this group showed the lowest yield of all vaccination groups. In fact, overall the bacterial counts were highly variable within and between groups. These findings might be attributable to the generally highly variable efficacy of BCG, documented in both animals and humans, or to some extent due to animal to animal variation or exposure to NTM. It is noteworthy that although recovery of BCG was successful in more animals in the formalin-inactivated BCG and heat-killed *M*. *bovis* groups, the concentration of bacteria was lowest in the latter group. Furthermore, although no significant differences were found, the bacterial counts in the heat-killed *M*. *bovis* group were lower as compared to unvaccinated animals (estimate ratio = 0.73). In contrast, the bacterial counts in the live and formalin-inactivated BCG groups were higher. These findings suggest that live BCG was able to replicate to a lesser extent in animals vaccinated with heat-killed *M*. *bovis*.

### Low immunogenicity of BCG vaccine formulations

Although highly variable, vaccination with live BCG has previously been found to elicit cell-mediated immune responses in cattle and to some extent provide protection against bovine tuberculosis [42, 43]. In the present study, including it as benchmark vaccine, live BCG did not induce significant cell-mediated immune responses as compared to the control group. Relatively poor immunogenicity of the vaccine, in the sense of *M*. *bovis* directed CMI responsiveness, may have partly been caused by prior exposure to NTM [44, 45], which was reflected by the elevated PPD-A and PPD-F responses (Fig 2B and 2C). Interestingly, animals in the heat-killed *M*. *bovis* group were able to mount a cell-mediated immune response strongly biased towards *M*. *bovis*, possibly suggesting that prior exposure to NTM might not interfere with the response elicited by this inactivated vaccine formulation. If true, this could be of high importance for countries with high prevalences of both BTB and NTM, including South Africa [44, 46].

In the present study, the results found by Whelan et al. [16] could not be reproduced as cell-mediated and humoral immune responses detected in calves in the formalin-inactivated BCG group were similar to or below those in the control group. The discrepancy in findings may be due to the effects of certain critical variables on the study outcome rather than poor reproducibility and warrant further investigation. Variables which differed between the two studies included the use of a different adjuvant, the length of formalin treatment, the use of a different strain of BCG and/or disparate exposure to different environmental mycobacteria. Furthermore, as live BCG proved to show poor immunogenicity in the present study, it is to be expected that a killed preparation of the same strain would have a similar or lower immunogenicity.

### Limitations of the study

One of the limitations of this study was that challenge with virulent *M*. *bovis* was not possible and it is recommended that in future studies this is carried out in order to empirically assess pathological changes as a function of vaccine efficacy of the heat-killed *M*. *bovis* vaccine candidate.

The use of multiple statistical models increases the chance of observing a significant result. However, we believe that the significant results described in this study are not only statistically but also biologically relevant. The wide confidence intervals that were found in several analyses carried out in this study, can be explained by the fact that a relatively low number of animals was used, which was the main limitation of the current study. Nevertheless, strong evidence for a high immunogenicity of the heat-killed *M*. *bovis* vaccine was demonstrated which justifies further vaccine trials to evaluate its usefulness in future BTB control strategies.

Lastly, our results indicate that there was exposure to environmental mycobacteria during the course of the experiment, as demonstrated by the responses to PPD-A and PPD-F in the BOVIGAM^®^ assay, as well as PPD-A reactivity in the skin test. The true *M*. *bovis* specific effects of vaccination might be masked by this responsivity. However, it is important to note that non-tuberculous mycobacteria occur ubiquitously in the environment on a global scale. Therefore, it is promising that the heat-killed *M*. *bovis* vaccine performed well under these circumstances, as they should closely resemble field conditions.

## Conclusion

The results obtained in the present study clearly indicate that subcutaneous vaccination with the heat-killed *Mycobacterium bovis* vaccine elicits strong and sustained cell-mediated and humoral immune responses in cattle, indicating excellent immunogenicity of the vaccine. Although exact correlates of protection are not known and this remains an important point of discussion between research groups, it may be assumed that an IFN-γ response such as demonstrated in this study, forms part of the protective immune profile against BTB. The role of humoral immunity in providing protection against BTB is even less well established, but more evidence is becoming available suggesting its value. Therefore, the finding of both strong CMI and HI responses to the heat-killed *M*. *bovis* vaccine, in combination with a lesser degree of *M*. *bovis* BCG replication after challenge, highlights the potential of this vaccine candidate. In further studies, challenge with virulent *M*. *bovis* is recommended as well as assessment of the vaccine in field studies to further evaluate vaccine efficacy under natural conditions.

## Supporting information

S1 Fig Conventional PCR for the detection of *Mycobacterium bovis*. PCR targeting RD1, RD4 and RD9 as previously described. PCR products of +- 268bp (RD4 absent), +- 196bp (RD1 absent) and +- 108bp (RD9 absent) indicate *M*. *bovis* BCG. Animals 18, 21 and 31 belong to group 1 (live *M*. *bovis* BCG), animals 2, 6, 7, 16 and 29 belong to group 2 (formalin-inactivated *M*. *bovis* BCG), animals 8, 9, 10, 11 and 26 belong to group 3 (heat-killed *M*. *bovis*) and animals 12 and 15 belong to group 4 (control). R = right prescapular lymph node.
(TIF)

S1 Dataset Tables containing the raw data of the immunological assays. (A) BOVIGAM assay. OD-values for all stimulations and controls. (B) IDEXX TB ELISA. OD-values for the samples and controls as well as S/P-ratio. (C) Skin test. Skin fold thickness measurements at 0hrs, 72hrs and the difference (Δmm) in mm. Avian = PPD-A; Bovine = PPD-B; PC1 = protein cocktail 1; PC2 = protein cocktail 2. (D) Culture. Weights (g) and bacterial counts (CFU/g of PLN) of left and right PLNs.
(XLSX)

S2 Dataset Tables describing the statistical models and their outcomes. (A) Linear mixed effects models describing PPD-B and the ratios of PPD-B/PPD-A and PPD-B/PPD-F. Outcome = a + b1 * time + b2 * group + b3 * (time * group). Data were log transformed in order to meet the model assumptions of normality and homoscedasticity. Back-transformed estimates and 95% confidence intervals are given. Significant results are in bold. (B) Linear mixed effects models describing ESAT-6 and CFP-10. Outcome = a + b1 * time + b2 * group + b3 * (time * group). Data were log transformed in order to meet the model assumptions of normality and homoscedasticity. Back-transformed estimates and 95% confidence are given. Significant results are in bold. (C) Linear mixed effects model describing the S/P ratio. Outcome = a + b1 * time + b2 * group + b3 * (time * group). Data were log transformed in order to meet the model assumptions of normality and homoscedasticity. Back-transformed estimates and 95% confidence intervals are given. Significant results are in bold. (D) Double generalized linear model describing ΔPPDB—ΔPPDA in the skin test. Outcome = a + b1 * group. Estimates and 95% confidence intervals are given. Significant results are in bold. (E) A simple general linear model describing ΔPC1 and ΔPC2. Outcome = a + b1 * group. Estimates and 95% confidence intervals are given. Significant results are in bold. (F) Linear mixed effects model describing the PLN weights. Outcome = a + b1 * LN side + b2 * group + b3 * (LN side * group) + b4 * gender. Data (PLN weights) were log transformed in order to meet the model assumptions of normality and homoscedasticity. Estimates and 95% confidence intervals are given. Significant results are in bold. (G) Negative binomial generalized linear model describing the bacterial counts. Outcome = a + b1 * group. Back-transformed estimates and 95% confidence intervals are given. Significant results are in bold.
(XLSX)
