# Supplementary figures and images for: Immune response profiles of calves following vaccination with live BCG and inactivated Mycobacterium bovis vaccine candidates
Source: PLoS One. 2017 Nov 20;12(11):e0188448. doi: 10.1371/journal.pone.0188448 (PMC5695775; doi:10.1371/journal.pone.0188448)

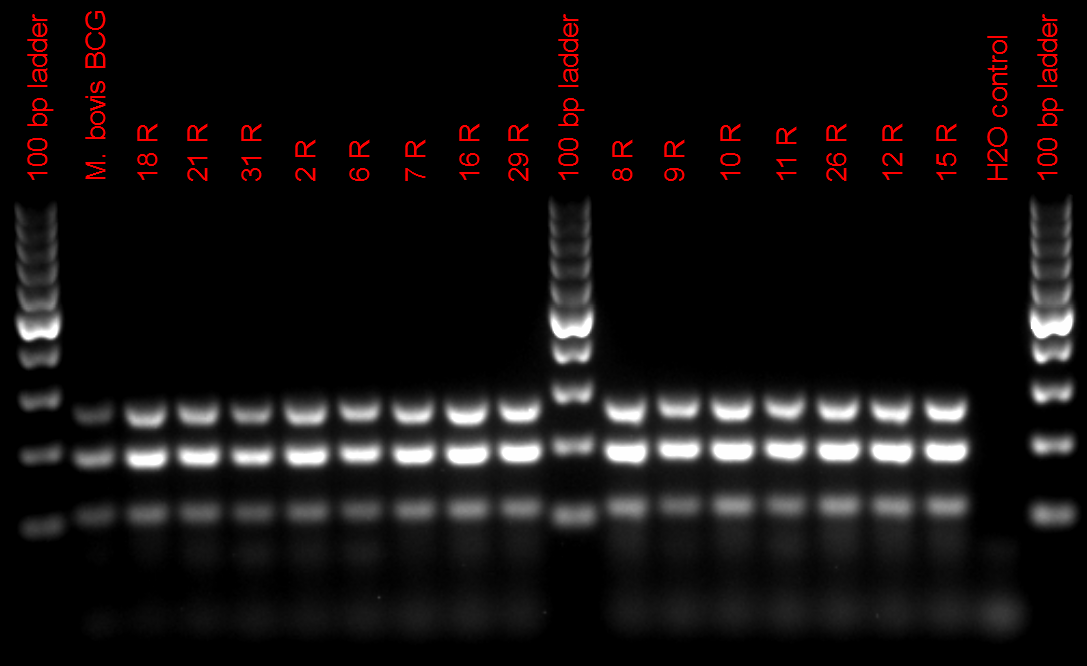

Supplement: S1 Fig — PCR targeting RD1, RD4 and RD9 as previously described. PCR products of +- 268bp (RD4 absent), +- 196bp (RD1 absent) and +- 108bp (RD9 absent) indicate M. bovis BCG. Animals 18, 21 and 31 belong to group 1 (live M. bovis BCG), animals 2, 6, 7, 16 and 29 belong to group 2 (formalin-inactivated M. bovis BCG), animals 8, 9, 10, 11 and 26 belong to group 3 (heat-killed M. bovis) and animals 12 and 15 belong to group 4 (control). R = right prescapular lymph node. (TIF) [file pone.0188448.s001.tif]
